# Medical students’ knowledge and practices regarding skin cancer and climate change-related dermatological risks: a cross-sectional study from Turkey

**DOI:** 10.1136/bmjopen-2025-110670

**Published:** 2025-12-30

**Authors:** Ece Karaoglu, Burcu Kucuk Bicer

**Affiliations:** 1 Gazi University Faculty of Medicine, Ankara, Turkey; 2 Medical Education and Informatics, Gazi University, Ankara, Turkey

**Keywords:** Climate Change, Education, Medical, Cancer

## Abstract

**Objective:**

Skin cancer represents one of the most preventable yet rapidly increasing malignancies worldwide, with projected rises associated with climate change. This study aimed to assess medical students’ knowledge, attitudes and practices regarding skin cancer and climate-related dermatological risks, and to identify demographic and educational predictors of awareness and preventive behaviours.

**Design:**

Cross-sectional survey.

**Setting:**

Public university medical faculty in Turkey.

**Participants:**

A total of 622 medical students enrolled in all six academic years completed the online questionnaire. Inclusion criteria were current enrolment and voluntary participation; incomplete submissions were excluded.

**Primary and secondary outcome measures:**

Primary outcomes were Skin Cancer Knowledge (SCKS) and Climate Change Knowledge (CCKS) Scores. Secondary outcomes included students’ perceived risk and photoprotective behaviours.

**Results:**

Mean SCKS was 7.81±3.06 and mean CCKS was 12.27±3.67. Female students had significantly higher SCKS (β=0.58; p<0.001) and CCKS (β=0.41; p<0.001). Although 92.3% recognised peak ultraviolet hazard hours, only 53.2% avoided midday exposure. A total of 64.1% reported at least one lifetime sunburn. Logistic regression showed that gender (OR=2.56; 95% CI 1.73 to 3.80), academic year (eg, Yr1 vs Yr6 OR=0.41; 95% CI 0.22 to 0.78), poor self-assessed knowledge (OR=3.19; 95% CI 1.33 to 7.64) and CCKS (per-unit increase, OR=0.92; 95% CI 0.87 to 0.96) significantly predicted perceiving climate change as a health threat.

**Conclusions:**

Medical students demonstrated substantial knowledge gaps and behavioural inconsistencies regarding skin cancer and climate-related dermatologic risks. Findings highlight the urgent need for structured, behaviourally oriented, climate-integrated dermatology education within medical curricula.

**STRENGTHS AND LIMITATIONS OF THIS STUDY**

- The study used a validated and pretested instrument to assess knowledge, attitudes and behaviours regarding skin cancer and climate change.
- The study included participants from all six academic years to enable comparisons across different stages of medical education.
- Data collection relied on self-reported online questionnaires, which may introduce recall and social desirability bias.
- The study’s cross-sectional design precludes causal inference between knowledge, attitudes and preventive behaviours.
- Convenience sampling from a single institution may reduce the generalisability of the findings.

## Introduction

 Skin cancer represents one of the most prevalent forms of cancer globally, with over 1.5 million newly diagnosed cases reported in 2022 alone.^1^ While non-melanoma skin cancers are more common, malignant melanoma remains the primary contributor to skin cancer-related mortality due to its aggressive nature and metastatic potential.^2^ Among the modifiable risk factors, ultraviolet (UV) radiation exposure is universally acknowledged as the leading environmental cause of skin carcinogenesis. Notably, the changing global climate has been increasingly implicated in modifying UV radiation exposure levels, thereby contributing to an elevated incidence of UV-related skin malignancies.^3^

A longitudinal analysis of the Global Burden of Disease Study (1990–2017) revealed a staggering 310% increase in the incidence of cutaneous squamous cell carcinoma, marking it as the fastest-growing cancer type within the same timeframe.^4^ Such trends are paralleled by a substantial rise in healthcare expenditures. For instance, while the costs associated with most cancers rose by approximately 25.1% between 2002 and 2011, skin cancer-related costs surged by 126.2%.^5^ These data highlight the urgent need for effective and sustainable public health strategies to curb this escalating burden.

Prevention strategies are typically stratified into three tiers, with primary prevention focusing on reducing UV exposure through behavioural interventions, and secondary prevention aiming at early detection through regular skin examinations.^6 7^ Primary care providers occupy a unique position in implementing these strategies due to their frontline role in patient education and early diagnosis.^8^

In this regard, medical students, as future healthcare professionals and potential advocates for climate-responsive public health policies, must be adequately trained in skin cancer prevention and early detection. However, studies consistently report deficiencies in medical students’ knowledge regarding UV exposure, sunscreen use and preventive behaviours, despite recognising the general importance of skin cancer.^9^ Furthermore, the dermatology component in medical school curricula remains limited, creating a gap between knowledge acquisition and preventive practice.^10 11^ Educational interventions, although few, have demonstrated promising outcomes in improving students’ competencies in this domain.^12 13^

It is important to emphasise that the dermatological impacts of climate change are not limited to skin cancer alone. Rising global temperatures and environmental alterations have been linked to increased incidence of inflammatory and chronic dermatoses, skin infections, vector-borne diseases and exacerbation of chronic skin conditions.^14^

Despite the increasing incidence and preventability of skin cancer, national studies focusing on medical students’ knowledge, attitudes and behaviours in Turkey remain limited in both scope and number.^15^,^18^ Our study offers a unique contribution by evaluating students across all academic years and incorporating their perceptions of the dermatological impacts of climate change.

Therefore, this study aims to assess the knowledge, attitudes and behaviours of Turkish medical students regarding UV radiation and skin cancer, as well as their perceptions of climate change and its effects on skin health, with the goal of providing empirical evidence to inform future educational and public health interventions.

## Methods

### Participants and study design

A single-centre, cross-sectional design was implemented.

Gazi University Faculty of Medicine is one of Turkey’s large and established state medical schools and shares a student profile that is broadly representative of Turkish medical students. Moreover, medical schools across the country, including Gazi University, use the nationally mandated National Core Education Programme (Ulusal Çekirdek Eğitim Programı), and accredited faculties follow highly standardised curricula and competency frameworks. Because accredited medical schools in Turkey teach comparable syllabi, learning objectives and core dermatology content, the educational environment at Gazi is aligned with that of other accredited institutions nationwide. For this reason, although the study was conducted in a single centre, extrapolating broad educational patterns to Turkish medical students is reasonable, and generalisation should be interpreted with this curricular homogeneity in mind.

The target population comprised all actively enrolled medical students (N=1500) across six academic years at a single public medical faculty during the study period. This sampling frame represented the full institutional student body with no programme exclusions.

A convenience sampling strategy was used because it permits efficient online data collection from large student groups and is widely applied in educational and behavioural health research. While this approach supports feasibility and comprehensive access, it may introduce selection bias and limit representativeness; these issues are acknowledged in the Limitations section.

In this cross-sectional study, the required sample size for multivariable logistic regression analysis was determined a priori based on both methodological considerations and statistical power requirements. In logistic regression modelling, it is recommended to have a minimum of 10–15 outcome events per independent variable to ensure model stability and to reduce the risk of overfitting.

According to the study objectives and conceptual framework, 20 independent variables were planned to be included in the multivariable logistic regression model. Using a more conservative criterion of 30 participants per independent variable, the minimum required sample size was estimated as 600 participants.

In addition, a formal statistical power analysis was performed using G*Power software (V.3.1.9.7, Heinrich-Heine-Universität Düsseldorf, Germany). For the logistic regression model, a medium effect size (Cohen’s f²=0.15), a two-tailed alpha level of 0.05, and a desired statistical power of 90% (1−β=0.90) were assumed. Based on these parameters, the estimated minimum sample size ranged between 590 and 610 participants, depending on the number of predictors retained in the final model.

To account for a potential 5% non-response rate and missing data, the target sample size was increased to 630 participants. Ultimately, data from 622 participants were included in the final analysis, which exceeded the minimum required sample size determined both by the logistic regression modelling approach and the a priori power analysis.

No formal quotas were applied; however, recruitment announcements were uniformly distributed across all academic years to encourage balanced participation.

Inclusion criteria were: (1) active enrolment, (2) provision of electronic informed consent and (3) completion of all required survey items. Exclusion criteria included incomplete responses and duplicate submissions.

Convenience sampling may allow self-selection and social desirability bias. To mitigate these risks, participation was anonymous, voluntary and restricted to one submission per user. Nevertheless, geographic, sociocultural or institutional differences may influence behaviours, and future multicentre studies with stratified random sampling would further strengthen external validity. These considerations are addressed in the Discussion.

### Data collection

Two researchers performed a literature review to develop knowledge scales for both climate change and skin cancer. The survey was administered electronically via Google Forms between 15 June and 10 October 2024. Items were derived primarily from multiple reputable sources. Several items were directly adapted from the validated Skin Cancer and Sun Knowledge Scale.^19^ Additional questions were developed by the research team after reviewing systematic reviews^9^ and studies among medical students on skin cancer knowledge,^20^ as well as international guidelines^2^ and recent literature on the dermatological impacts of climate change.^14 21^ Instrument reliability and internal consistency were confirmed through a pilot study. Cronbach’s alpha coefficients were 0.917 for the Climate Change Knowledge Score (CCKS) and 0.716 for the SCKS, indicating high and acceptable reliability, respectively.

Survey invitations containing the study information sheet and a secure questionnaire link were disseminated to all eligible students via official institutional email channels. The invitation emphasised voluntary participation, anonymity and withdrawal options; no incentives were provided. Electronic informed consent was obtained from all participants. The survey platform prevented duplicate responses.

Although messages were sent to all students, prior research shows that institutional emails are not always viewed. Published estimates indicate that roughly 10%–20% of institutional emails remain unopened, consistent with average response rates of ~44% reported in higher-education online surveys. Accordingly, we conservatively estimate that approximately 1200–1350 students likely viewed the invitation. This permits more accurate participation denominator reporting and aligns with recommended transparency in online recruitment.

**Figure 1:**
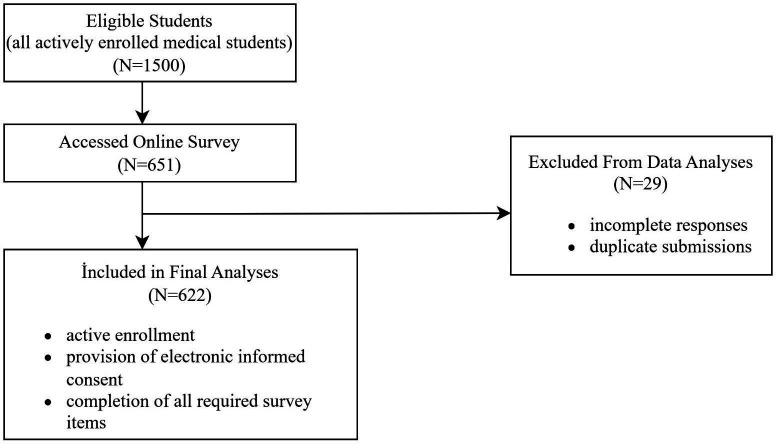
Participant flow diagram.

Among these, 651 students accessed the survey, and after excluding 29 incomplete submissions, 622 students were retained for analysis (see figure 1), yielding an estimated completion rate of 46.1%–51.8% among those who likely viewed the invitation. Students from all six academic years participated, providing broad representation across educational strata.

### Measurement of variables

The final instrument comprised 52 items across five sections:

Demographic and general characteristics (see table 1).

Skin cancer knowledge, risk factors, and prevention behaviours (score range: 0–18; see online supplemental table 1).

Climate change knowledge and associated skin health risks (score range: 0–15; see online supplemental table 2).

Self-reported sun-protective behaviours (eight items; see online supplemental table 3).

Each correct response was awarded one point. A total score of 10–18 was classified as high SCKS. Similarly, a CCKS between 8 and 15 was considered indicative of good knowledge regarding climate change.

**Table 1:** Sociodemographic characteristics of participants stratified by gender

Sociodemographic characteristic	Male (%)	Female (%)	Total (%)	P value
Academic year				
Clinical students	45.1	50.4	48.4	0.447
Preclinical students	54.9	49.6	51.6	
Socioeconomic status				
High	32.2	35.2	34.1	0.206
Middle–low	63.1	59.6	60.9	

### Demographic and general characteristics

Demographic variables included gender, year of study (preclinical or clinical), and socioeconomic level. Additionally, participants were queried regarding personal or familial history of skin cancer, information sources and Fitzpatrick skin type. Skin type was self-assessed using descriptive statements aligned with the six-point Fitzpatrick classification (type I–VI). Likert-scale questions (Agree-Disagree-No Idea) were employed for several knowledge-based items.

### Data analysis

Descriptive statistics (means and SD) were used for continuous variables, and frequencies and percentages were used for categorical variables. Given that sample sizes exceeded 30 in all subgroups, the central limit theorem supported the use of parametric tests. Independent samples t-tests were employed to compare continuous variables between groups. Pearson correlation analysis was conducted to examine relationships between knowledge, attitudes and practices (KAP) regarding climate change and skin cancer.

Binary logistic regression analysis was performed to identify predictors of viewing climate change as a health threat. Variables with p values less than 0.20 in univariate analysis were entered into the multivariate logistic regression model using a backward conditional approach. All analyses were carried out using SPSS V.25.0 (IBM), with p values less than 0.05 considered statistically significant.

## Results

Medical students from all academic years participated in the study (n=622). The distribution across academic years was relatively balanced: 20.4% were first-year, 16.4% second-year, 14.8% third-year, 15.8% fourth-year, 16.2% fifth-year and 16.4% sixth-year students. Among the participants, 62.5% were female and 37.5% were male. No statistically significant differences were observed in the distribution of students across clinical/preclinical years or socioeconomic levels by gender (p>0.05) (table 1).

Only 4.2% (n=26) of respondents reported a personal history of skin cancer. A substantial proportion of participants (86.3%) reported not knowing their skin type. However, when descriptions based on the Fitzpatrick scale were provided, female students were significantly more likely to identify their skin type compared with males (p<0.05) (table 2).

**Table 2:** Gender-based differences in Fitzpatrick skin types and Skin Cancer Knowledge Score

	Male (%)	Female (%)	Total (%)	P value
Fitzpatrick skin type				
Type 1	1.7	1.8	1.8	0.206
Type 2	1.3	5.1	3.7	
Type 3	4.3	5.9	5.3	
Type 4	2.6	2.6	2.6	
Type 5	0.4	0.3	0.3	
Does not know	**89.7**	**84.3**	**86.3**	
Fitzpatrick skin type				
Always burns, never tans	1.3	4.1	3.1	**<0.01**
Usually burns, tans minimally	11.2	22.1	18.0	
Sometimes burns, tans gradually	18.5	25.2	22.7	
Rarely burns, tans easily	16.7	21.6	19.8	
Never burns	1.7	2.3	2.1	
Does not know	**50.6**	**24.7**	**34.4**	
Skin Cancer Knowledge Score	7.04±3.2	12.98±2.8	7.82±3.1	**<0.001**

Bold values indicate statistically significant results (p<0.05) and the predominant response category.

### Knowledge findings

The mean SCKS was 7.81 (SD=3.06; range=0–16). Knowledge regarding skin cancer and UV radiation was generally low. Only 17.7% correctly identified basal cell carcinoma (BCC) as the most common skin cancer, and 34.7% correctly named melanoma as the deadliest form. Regarding melanoma, only 49.2% of students correctly identified that having numerous melanocytic nevi is a risk factor for skin cancer. Meanwhile, 46.9% correctly rejected the false notion that melanoma develops solely from pre-existing nevi. These findings highlight a lack of awareness concerning the role of melanocytic nevi in melanoma development. Regarding UV radiation, 41% correctly identified UVA as the most prevalent form reaching Earth, and 29.1% recognised UVB as the type primarily responsible for skin cancer pathogenesis. While the majority understood that UV exposure remains a risk under cloudy conditions (81.8%) and over snow-covered surfaces (60.6%), misconceptions persisted regarding the reduced protection offered by light-coloured (13.3%) and wet clothing (20.6%). Moreover, only 36.2% knew the correct definition of SPF, and knowledge of UV types protected by SPF (10%) and PFA values (20.6%) was low.

In terms of CCKS, the average CCKS was 12.27 (SD=3.67). A low but significant positive correlation was found between CCKS and SCKS (r=0.218, p<0.001). The most commonly associated health effects were acute sunburn (92.3%), skin barrier disruption (91.5%), and increased risk of heat-related illnesses (91.5%). Less frequently recognised effects included immunosuppression (55.3%) and increase in vector-borne infectious diseases (65.8%). Female students had significantly higher mean CCKS (12.98±2.8) than males (11.09±4.6) (p<0.001), although male students were more likely to self-report a high level of CCKS (p=0.013). Notably, women were more likely to perceive climate change as a serious health threat (p=0.001) (table 3).

**Table 3:** Gender-based differences in knowledge and perception of climate change

	Male (%)	Female (%)	Total (%)	P value
Self-assessed knowledge level on climate change				**0.013**
Good	11.6	4.6	7.2	
Moderate	50.2	56.0	53.9	
Poor	31.3	32.9	32.3	
No opinion	6.9	6.4	6.6	
Climate Change Knowledge Score	11.09±4.6	12.98±2.8	12.27±3.7	**<0.001**
Perception of climate change as a health threat	58.4	79.4	71.5	**0.001**

Bold values indicate statistically significant results (p<0.05).

### Behavioural findings

**Figure 2:**
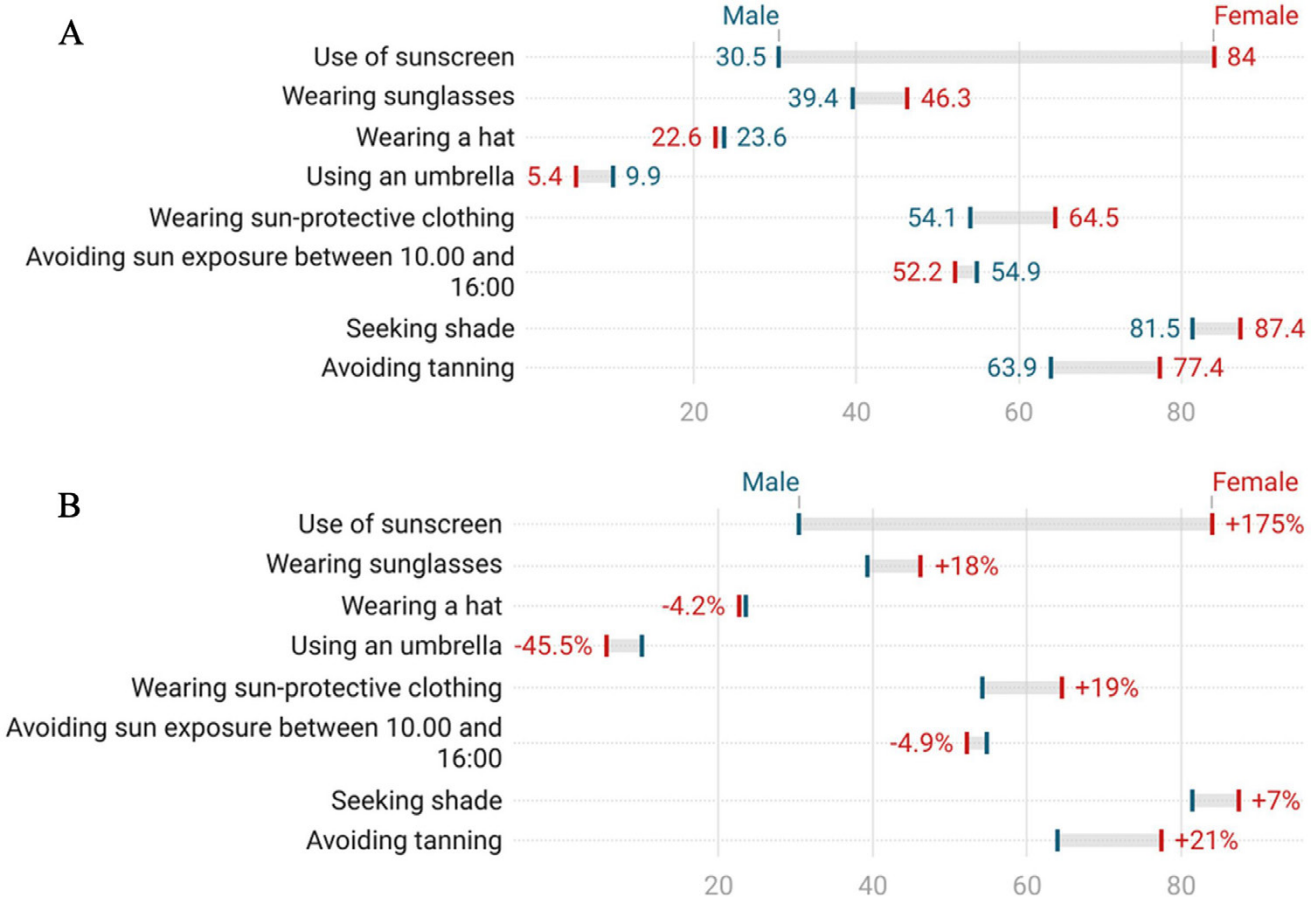
Sun protection behaviours by gender. (A) Percentages of ‘always’ responses for sun protection behaviours by gender. (B) Gender-based percentage differences in ‘always’ responses for sun protection behaviours.

According to the proportion of ‘always’ responses, avoiding tanning (43.9%) and seeking shade (43.7%) were the most common sun-protective behaviours, followed by sunscreen use (41.5%) and wearing protective clothing (26.7%). In contrast, hat (8.5%) and umbrella use (3.4%) were the least common (figure 2A).

Sun-protective behaviours were generally more prevalent among female students. The largest gender difference was observed in sunscreen use, which was approximately 175% higher among females. Similar differences were found in avoiding tanning (21% higher) and the use of sun-protective clothing (19% higher). Conversely, although the overall prevalence remained low, umbrella use (45.5% higher) and avoiding sun exposure during midday (4.9% higher) were more common among male students. These relative differences were calculated using male percentages as the reference point (figure 2B).

Although the majority of students (92.3%) correctly identified the most hazardous time of day for sun exposure, only 53.2% reported avoiding the sun during this interval. This discrepancy is a striking example of knowledge not translating into preventive behaviour.

Among sunscreen users, only 45.3% reported regular use throughout the year, and 58.5% did not reapply sunscreen during the day. The most cited reasons for non-use were lack of habit (38.6%), high cost (20.8%) and forgetfulness (14.7%). Furthermore, 28% of students had never heard of the UV Index, and 48.1% did not check it before going outdoors. Widespread misconceptions about sun protection were also detected. For example, 68.2% of students believed that safe tanning is possible with sunscreen, and 42% believed that gradual tanning eliminates harmful effects. Interestingly, while 59.2% of students reported never sunbathing for tanning purposes, 64.1% had experienced at least one sunburn in their lifetime.

### Perceptions of climate change: multivariate analysis

Logistic regression analysis revealed that gender, year of study, self-reported knowledge and CCKS were significant predictors of perceiving climate change as a health threat (p<0.001). Female students, preclinical students, those with higher CCKS and those with lower self-reported CCKS were more likely to perceive climate change as a health threat (table 4). These findings underscore the importance of knowledge level and perceived self-efficacy in shaping threat perception.

**Table 4:** Factors associated with the perception of climate change as a health threat: multivariate logistic regression analysis

Perception of climate change as a health threat	P value	OR (%95 CI)
Constant	0.051	
Gender		
Female		R
Male	**<0.001**	2.563 (1.727 to 0.803)
Academic year		
6		R
1	**0.006**	0.410 (0.217 to 0.775)
2	**0.001**	0.320 (0.161 to 0.635)
3	**0.003**	0.349 (0.173 to 0.704)
4	0.197	0.655 (0.344 to 0.246)
5	0.326	0.727 (0.384 to 0.375)
Self-assessed knowledge level on climate change		
Good		R
Moderate	0.337	1.524 (0.645 to 0.603)
Poor	**0.009**	3.189 (1.332 to 0.636)
No opinion	**0.006**	1.546 (1.546 to 3.242)
CCKS	**0.001**	0.916 (0.870 to 0.965)

Bold values indicate statistically significant results (p<0.05).

* Gender, socioeconomic status, academic year, skin type, personal/family history, SCKS, self-assessed knowledge level on climate change, CCKS.

† R2=76.2

CCKS, Climate Change Knowledge Score; R, Reference category; SCKS, Skin Cancer Knowledge Score.

While a greater proportion of preclinical students rated their knowledge level as ‘good’ (11.6%) compared with clinical students (4.6%) (p=0.013), actual CCKS was significantly higher among clinical students (p=0.007). Furthermore, the perception of climate change as a health threat was more prevalent among preclinical students (p=0.013). Although clinical students scored higher on SCKS, the difference did not reach statistical significance (p=0.091), though it may still be of clinical relevance (table 5).

**Table 5:** Climate change and skin cancer awareness and perceptions by academic level

Climate change and skin cancer	Preclinical students	Clinical students	Total	P value
Self-assessed knowledge level on climate change				
Good	11.6	4.6	7.2	**0.013**
Moderate	50.2	56.0	53.9	
Poor	31.3	32.9	32.3	
No opinion	6.9	6.4	6.6	
CCKS	11.83±3.8	12.74±3.5	12.27±3.7	**0.007**
Perception of climate change as a health threat	244 (76.0)	201 (66.8)	445 (71.5)	**0.013**
SCKS	6.3±2.5	9.4±2.8	7.82±3.1	**0.091**

Bold values indicate statistically significant results (p<0.05).

CCKS, Climate Change Knowledge Score; SCKS, Skin Cancer Knowledge Score.

Despite higher knowledge scores and greater engagement in photoprotective behaviours, female students reported higher frequencies of sunburn (p=0.008), suggesting a potential gap between awareness and effective implementation.

## Discussion

This study investigated medical students’ knowledge, attitudes and behaviours regarding the effects of climate change on skin health and skin cancer prevention. Our findings revealed suboptimal knowledge levels in both domains. When considered alongside the prevalence of misconceptions and inconsistent sun-protective behaviours, these results underscore substantial educational deficiencies. This pattern is consistent with previous national^15^,^18^ and international studies^9^,^1122 23^ that highlight inadequate dermatological education among medical students.

One notable observation was that the majority of students were unable to identify their skin type without descriptive assistance, reflecting a low level of personal risk awareness. This is particularly concerning, as skin type is a key determinant of susceptibility to UV-induced skin damage and skin cancer.^6^ Although 59.2% of students stated they did not intentionally tan, 64.1% reported having experienced at least one sunburn. Supporting these findings, while 89.9% were aware that sunscreen use alone is insufficient for skin cancer prevention, many still failed to adopt additional photoprotective strategies such as wearing protective clothing, hats or using umbrellas, consistent with findings in the literature.^9^ This suggests that photoprotection strategies are often poorly implemented or occur passively and without conscious risk assessment.

Similar deficiencies in knowledge and protective behaviours have been documented in studies involving primary care physicians in Turkey. Conversely, the same study found that physicians who personally used sunscreen were significantly more likely to recommend it to their patients compared with those who did not.^24^ These results reveal a disconnect between risk perception, preventive intent and behavioural implementation, even among health-literate individuals. This discrepancy is further illustrated by a study comparing dermatology patients, medical students and general practitioners: although students and physicians had higher knowledge and attitude scores, patients demonstrated significantly better protective practices.^11^

Knowledge deficiencies regarding skin cancer subtypes (eg, BCC and malignant melanoma) and UV radiation (UVA, UVB) were also apparent and mirrored international findings.^9 10^ While 77.8% of participants acknowledged the potential link between climate change and an increased incidence of cutaneous malignancies, only 34.7% correctly identified melanoma as the most fatal form of skin cancer. This may reflect the relatively low concern students express toward skin cancer compared with other cancers.^9^ Furthermore, only 49.2% recognised the number of melanocytic nevi as a risk factor, and just 46.9% knew that melanomas can arise de novo. These results point to insufficient understanding of melanoma pathogenesis, a gap also identified in a national study by Kaya İslamoğlu *et al*.^15^

Behavioural deficits in photoprotection appear to correlate with deficiencies in UV-related knowledge. Only about 30% of students correctly identified UVB as the main radiation responsible for skin carcinogenesis. Fewer than half accurately understood the definition of sun protection factor (SPF) and the types of UV radiation (UVA vs UVB) covered by SPF/protection factor of UVA (PFA) ratings. Roughly half of the participants had never checked the UV Index, suggesting a lack of awareness of how to assess UV exposure risk and tailor protection accordingly. Similarly, a study among medical university students in Northeast China reported that knowledge of UVR-related health risks and understanding of the UV Index were not comprehensive, and that sun-protective behaviours remained limited.^25^ Although approximately 64.4% of students reported always or frequently using sunscreen, this behaviour may often be habitual rather than informed. These findings are consistent with Compres *et al*,^12^ who reported that short-term dermatology interventions significantly enhance both knowledge and preventive behaviour among medical students.

Pronounced gender differences were observed. Female students achieved significantly higher scores on both the CCKS and SCKS, were more likely to engage in photoprotective behaviours, and more frequently perceived climate change as a serious health threat (p<0.001). Paradoxically, despite greater awareness, female students also reported higher rates of sunburn. This may suggest that knowledge does not always mitigate risk behaviour, possibly due to aesthetic or cultural motivations related to tanning, as highlighted by Nahar *et al*.^9 11^

The academic year was also associated with differences in knowledge perception. Clinical-year students had significantly higher objective knowledge scores (CCKS and SCKS) compared with preclinical students but tended to rate their knowledge lower. This mismatch may reflect greater realism in clinical students’ self-assessment or increasing recognition of complexity as training advances. Conversely, preclinical students were more likely to overestimate their knowledge, indicating the need for early, accurate feedback mechanisms in medical education.^11 13^

Multivariate regression analysis revealed that gender, academic year and both perceived and objective knowledge levels significantly influenced students’ perception of climate change as a health threat. Students with greater objective knowledge and more modest self-assessments were more likely to express concern about climate-related health risks. This is in line with previous studies linking health literacy with enhanced risk perception.^8 11^

A recent study involving family physicians in Turkey found that nearly 60% had encountered patients with suspected skin cancer and referred them for further evaluation.^24^ However, despite a similar proportion of patients consulting physicians about sunscreen use, fewer than half of physicians reported recommending sunscreen to high-risk individuals. This indicates that although primary care encounters with skin cancer are frequent, significant gaps persist in both knowledge and the implementation of preventive counselling.

This study demonstrates that medical students show substantial gaps in knowledge, risk perception and preventive behaviours regarding UV radiation, skin cancer and the dermatological impacts of climate change. We determined partial awareness of the association between climate change and increasing skin cancer risk; however, students displayed limited understanding of UV-related mechanisms, melanoma risk factors and evidence-based photoprotection strategies. Furthermore, the misalignment between students’ knowledge and their actual preventive practices suggests that current educational strategies fall short in fostering sustained competence and meaningful behavioural change, a gap that may be partly explained by prevailing sociocultural norms.

To address these limitations, integration of a spiral curriculum model into undergraduate medical education is strongly recommended. A spiral design, in which key concepts are revisited and expanded across different stages of learning, would allow progressive reinforcement of foundational knowledge on UV radiation, skin carcinogenesis and climate-sensitive health risks. Introducing these concepts in early medical education through basic sciences and public health modules, and reinforcing them in later years through clinical dermatology, patient communication and community-based preventive practices, would support deeper understanding and sustainable skill development. Longitudinal educational interventions incorporating behavioural and attitudinal components may further foster consistent professional and personal photoprotective practices among future physicians.

In conclusion, embedding climate-related skin health, UV radiation and skin cancer prevention within a spiral curriculum framework may represent an effective and sustainable educational strategy to improve medical students’ preparedness for emerging climate-related health challenges. Such curricular refinement may play a critical role in developing physicians who are not only knowledgeable, but also capable of translating this knowledge into preventive clinical and public health action.

### Limitations

As data were collected from a single institution using convenience sampling, findings may not be generalisable to medical students in other regions, where dermatology curricula, sun-exposure norms and climate-risk perceptions differ. Previous KAP studies have noted substantial contextual variation across geographic and sociocultural settings, including UV index intensity, tanning norms and sunscreen affordability. This underscores the need for multicentre research across diverse educational and climatic environments.

Reliance on self-reported behaviours may have inflated photoprotective practices or underestimated harmful exposures. Recall bias and social-desirability pressures are well-documented limitations in KAP research involving preventive health behaviours. Objective assessment, such as clinical skin type characterisation, UV-monitoring wearables, would strengthen future studies.

Although the CCKS displayed strong internal consistency, it was newly developed; thus, comparability to previously validated tools is limited. Nonetheless, its development was grounded in authoritative guidelines and recent synthesis of climate-dermatology evidence, suggesting reasonable content validity. Further validation, factor structure analysis and test–retest reliability remain an important next step.

Factors including cultural beauty norms, tanning behaviours, seasonal timing, latitude, income and dermatology exposure were not systematically assessed but may modulate photoprotective behaviour. Future studies should incorporate structured environmental and sociocultural determinants.

While binary logistic regression provided an interpretable framework for estimating predictors of perceived climate-related health risk, we also evaluated whether more complex modelling strategies would improve explanatory performance. Specifically, we examined potential interaction effects, including gender and academic year, to explore whether educational progression modified gender-based differences in risk perception. Interaction terms were entered into the model individually; however, none reached statistical significance (all p>0.05) and did not meaningfully improve model fit. Consequently, interaction terms were removed from the final multivariable model to preserve parsimony and interpretability.

Ordinal (or multinomial) models were additionally considered as alternative approaches to capture finer gradations in perceived risk. However, the distribution of the outcome variable and comparative fit statistics did not indicate clear advantages over the binary specification, and ordinal modelling did not materially change effect direction or robustness. Therefore, the binary logistic regression model was retained as the most parsimonious and interpretable representation of the data.

## Supplementary material

10.1136/bmjopen-2025-110670 online supplemental file 1

## Data Availability

All data relevant to the study are included in the article or uploaded as supplementary information.
