# Supplementary material for: Medical students’ knowledge and practices regarding skin cancer and climate change-related dermatological risks: a cross-sectional study from Turkey
Source: BMJ Open. 2025 Dec 30;15(12):e110670. doi: 10.1136/bmjopen-2025-110670 (PMC12766811; doi:10.1136/bmjopen-2025-110670)
Supplement: online supplemental file 1 [file bmjopen-15-12-s001.docx]

**Suppelementary Table**

**Supplementary Table 1.** Skin cancer knowledge, risk factors, and prevention behaviors (score range: 0–18)

The Skin Cancer Knowledge Score (SCKS) was based on the following questions.

Items

1. What type of UV radiation reaches the Earth most?

1. **UVA**
2. UVB
3. UVC
4. I don’t know

2. What type of UV radiation is most linked to skin cancer?

1. UVA
2. **UVB**
3. UVC
4. I don’t know

3. What is the most common form of skin cancer?

1. Squamous cell carcinoma
2. **Basal cell carcinoma**
3. Melanoma
4. I don’t know

4. What type of skin cancer has the highest mortality rate?

1. Squamous cell carcinoma
2. Basal cell carcinoma
3. **Melanoma**
4. I don’t know

5. Having numerous melanocytic nevi is a risk factor for skin cancer.

1. **True**
2. False
3. I don’t know

6. Melanoma develops only from pre-existing melanocytic nevi.

1. True
2. **False**
3. I don’t know

7. Which of the following time intervals is considered the most dangerous for sun exposure?

1. 9:00 AM – 12:00 PM
2. **10:00 AM – 4:00 PM**
3. 2:00 PM – 6:00 PM
4. 4:00 PM – 8:00 PM

8. Tanned skin is not affected by the harmful effects of sunlight.

1. True
2. **False**
3. I don’t know

9. Gradual tanning significantly reduces the harmful effects of prolonged sun exposure.

1. True
2. **False**
3. I don’t know

10. It is safe to get a tan while wearing sunscreen.

1. True
2. **False**
3. I don’t know

11. Which of the following best describes the protection provided by SPF 30 sunscreen?

1. **It means a person can stay in the sun 30 times longer without getting sunburn than if they weren’t wearing sunscreen.**
2. It provides twice as much protection as sunscreen with SPF 15.
3. It blocks 30% of UV radiation.
4. All of the above

12. Using only sunscreen provides enough protection against skin cancer.

1. True
2. **False**
3. I don’t know

13. Which types of UV radiation does SPF protect against?

1. UVA
2. **UVB**
3. UVA & UVB
4. UVC
5. UVA, UVB & UVC

14. Which type of UV radiation is primarily measured by the PA rating system (Protection Grade of UVA)?

1. **UVA**
2. UVB
3. UVA & UVB
4. UVC
5. UVA, UVB & UVC

15. Lighter-colored clothing offers more UV protection than darker clothing.

1. True
2. **False**
3. I don’t know

16. Wet clothing provides less UV protection than dry clothing.

1. **True**
2. False
3. I don’t know

17. UV rays are not dangerous on cloudy days.

1. True
2. **False**
3. I don’t know

18. Snow can increase your exposure to UV radiation.

1. **True**
2. False
3. I don’t know

**Supplementary Table 2.** Climate change knowledge and associated health risks (score range: 0–15)

The Climate Change Knowledge Score (CCKS) score was based on the following 15 items related to the potential effects of climate change on skin health.

Items

1. Disruption of the skin barrier

Agree

Disagree

No idea

2. Alterations in the skin microbiome

Agree

Disagree

No idea

3. Increase in allergic skin diseases

Agree

Disagree

No idea

4. Increase in inflammatory skin diseases

Agree

Disagree

No idea

5. Increased exacerbation of existing skin conditions

Agree

Disagree

No idea

6. Increase in vector-borne infectious diseases

Agree

Disagree

No idea

7. Increase in skin infections

Agree

Disagree

No idea

8. Increase in soft tissue infections

Agree

Disagree

No idea

9. Increased risk of heat-related illnesses

Agree

Disagree

No idea

10. Increase in photosensitivity reactions

Agree

Disagree

No idea

11. Acute sunburns

Agree

Disagree

No idea

12. Increase in chronic photodermatitis cases

Agree

Disagree

No idea

13. Photoaging

Agree

Disagree

No idea

14. Immunosuppression

Agree

Disagree

No idea

15. Increased incidence of cutaneous malignancies

Agree

Disagree

No idea

**Supplementary Table 3.** Self-reported sun-protective behaviors (8 items)

The sun-protective behavior score was based on the following 8 items, rated on a 5-point Likert scale.

Items and Response Options

1. Use of sunscreen Always/Sometimes/Usually/Rarely/Never

2. Wearing sunglasses Always/Sometimes/Usually/Rarely/Never

3. Wearing a hat Always/Sometimes/Usually/Rarely/Never

4. Using a sun umbrella/parasol for shade when outdoors. Always/Sometimes/Usually/Rarely/Never

5. Wearing sun-protective clothing such as long-sleeved shirts and trousers Always/Sometimes/Usually/Rarely/Never

6. Avoiding sun exposure between 10:00 a.m. and 4:00 p.m. Always/Sometimes/Usually/Rarely/Never

7. Seeking shade while outdoors Always/Sometimes/Usually/Rarely/Never

8. Avoiding tanning Always/Sometimes/Usually/Rarely/Never

**Abbrevations:**

SCKS: Skin Cancer Knowledge Score

UV: Ultraviolet

SPF: Sun Protection Factor

PA: Protection Grade of UVA

CCKS: Climate Change Knowledge Score
