# Supplementary material for: Medical students’ knowledge and practices regarding skin cancer and climate change-related dermatological risks: a cross-sectional study from Turkey
Source: BMJ Open. 2025 Dec 30;15(12):e110670. doi: 10.1136/bmjopen-2025-110670 (PMC12766811; doi:10.1136/bmjopen-2025-110670)
Supplement: Supplementary file 2 [file bmjopen-2025-110670.reviewer_comments.pdf]

PEER REVIEW HISTORY

BMJ Open publishes all reviews undertaken for accepted manuscripts. Reviewers are asked to complete a checklist review form and are provided with free text boxes to elaborate on their assessment. These free text comments are reproduced below.

ARTICLE DETAILS

Title (Provisional)

Medical Students’ Knowledge and Practices Regarding Skin Cancer and Climate Change-Related Dermatological Risks: A Cross-Sectional Study from Turkey

Authors

Karaoglu, Ece; Kucuk Bicer, Burcu

VERSION 1 - REVIEW

Reviewer 1

Name Xu, Zhijie

Affiliation Zhejiang University School of Medicine Second Affiliated Hospital, Department of General Practice

Date 22-Oct-2025

COI None

This cross-sectional study addresses a timely and relevant topic. The topic is of significant educational importance, particularly in the context of rising skin cancer incidence and the evolving role of climate change in dermatological health. The authors have clearly articulated their objectives and have provided valuable insights into the gaps in medical education and student behavior. However, several methodological limitations warrant careful consideration, as they may affect the validity and generalizability of the findings.

First, The use of convenience sampling from a single medical school limits the representativeness of the sample. While the authors achieved a balanced distribution across academic years, the findings may not be generalizable to medical students in other regions of Turkey or in different educational contexts. A multi-center or stratified random sampling approach would have strengthened the external validity of the results.

Second, The reliance on self-reported measures for knowledge, attitudes, and behaviors introduces the possibility of recall bias and social desirability bias. For instance, students may overreport sun-protective behaviors or underreport risky practices. The lack of objective validation (such as direct observation of sunscreen use or clinical assessment of skin type) further limits the accuracy of behavioral data. Future studies could benefit from mixed-

method approaches to triangulate self-reported data with observational or clinical measures.

Third, although the survey demonstrated acceptable internal consistency, the climate change knowledge scale was newly developed and may lack established comparability with existing instruments. Additionally, the survey did not comprehensively assess external factors such as cultural attitudes toward tanning, seasonal variations in behavior, or socioeconomic barriers to sun protection, which could significantly influence the results. Including such variables would provide a more nuanced understanding of the determinants of photoprotective behavior.

Finally, while the statistical methods are generally appropriate, the use of a binary logistic regression model for a complex outcome like "perception of climate change as a health threat" may oversimplify the underlying construct. The model could have been enhanced by considering ordinal or multinomial logistic regression, or by including interaction terms to explore how gender and academic year jointly influence perception. Furthermore, the rationale for selecting variables for the multivariate model ($p < 0.20$ in univariate analysis) is not fully justified and may have led to the inclusion of non-predictive variables.

**Reviewer**	**2**
**Name**	**Hashizume, Hideo**
**Affiliation**	**Shimada Municipal Hospital**
**Date**	**23-Oct-2025**
**COI**	**None**

This is a timely and original manuscript that integrates climate health and skin-cancer prevention within a unified knowledge–attitudes–practices (KAP) framework. Nonetheless, external validity is uncertain. Cross-country differences in (i) medical-education structures and dermatology exposure, (ii) the curricular priority accorded to dermatology, and (iii) cultural norms around skin, sun exposure, and climate risk perception may yield heterogeneous results. To strengthen the paper, please:

Specify the target population and the settings to which the findings are intended to generalize.

Contextualize predictive factors and key outcomes—knowledge scores; behaviors (peak-UV avoidance, sunscreen reapplication, UV-index checking); and sex/gender differences—by benchmarking against representative studies from multiple regions.

Acknowledge potential unmeasured confounding (e.g., culture, season/latitude, socioeconomic position) and limitations of self-reported behaviors (recall and social-desirability bias).

Temper the conclusions as hypothesis-generating, outlining implications and testable next steps for climate-informed dermatology education across diverse contexts.

VERSION 1 - AUTHOR RESPONSE

REVIEWER 1

- **Comment R1.1 Single-center convenience sampling limits generalizability**

The use of convenience sampling from a single medical school limits representativeness.

Response:

We agree that convenience sampling may affect external validity. To address this, we expanded the Methods (Sampling and Recruitment) and Discussion to acknowledge this limitation and contextualize generalizability.

Importantly, Gazi University uses the nationally mandated National Core Curriculum (UÇEP) and shares accreditation standards and curricular structure with other accredited Turkish medical schools; therefore, educational content, dermatology exposure, and student profiles are broadly comparable nationwide. This provides contextual justification that our findings can reasonably reflect patterns among medical students in Turkey. Nonetheless, we emphasize this as a limitation and recommend future multicenter work.

Manuscript Change:

Methods → Participants and Study Design; pp. 5-6; lines 109-118.

Methods → Sampling and Recruitment; pp. 6; lines 122-125.

Discussion → Limitations; pp. 20; lines 395-400.

- **Comment R1.2 Self-reported behavior: recall/social desirability bias**

Response:

We acknowledge that self-reported behavior may introduce bias. We expanded the Discussion to emphasize possible over- or under-estimation of sun-protective behaviors and reinforced that anonymity was used to reduce social desirability bias. We explicitly highlight this in the Limitations.

Manuscript Change:

Discussion → Limitations; pp. 20; lines 401-405.

- **Comment R1.3 Novel CCKS scale considerations**

Response: The Discussion has been expanded to recognize that the CCKS is newly developed and that cross-study comparability may be limited. We note that internal consistency was good, and future psychometric validation is needed.

Manuscript Change:

Discussion → Limitations; pp. 20-21; lines 406-410.

- **Comment R1.4 Contextual factors not measured (culture, season, SES)**

Response: We agree. We added paragraphs acknowledging that sociocultural norms, seasonal factors, and socioeconomic conditions were not directly measured and could influence results. We recommend inclusion of these variables in future research.

Manuscript Change:

Discussion → Limitations; pp. 21; lines 411-414.

- **Comment R1.5 Logistic regression specification**

Response: We clarified the rationale for using binary logistic regression in the Limitations section, with reference to Babyak (2004) regarding the overfitting in regression-type models.

We justified the $p < 0.20$ threshold based on the purposeful selection approach described by Hosmer and Lemeshow.

We noted that interaction terms and ordinal models may be used in future studies.

Manuscript Change:

Discussion→Limitations; pp. 21; lines 415-428.

REVIEWER 2

- **Comment R2.1 — Clarify target population & generalizability**

Response:

We clarified the target population in Methods. To address generalizability, we added justification emphasizing that Gazi University represents a large Turkish medical faculty that follows the standardized National Core Education Program (UÇEP), similar to other accredited institutions in Turkey. Because medical curricula, learning objectives, and dermatology exposure are aligned nationwide, cautious extrapolation is reasonable. We still acknowledge single-center design as a limitation.

Manuscript Change:

Methods → Participants and Study Design; pp. 5-6; lines 109-118.

Discussion → Limitations; pp. 20; lines 395-400.

- **Comment R2.2 Benchmark outcomes against other regions**

Response:

We strengthened the Discussion by comparing our findings with international studies addressing dermatology-related KAP, photoprotection, and climate-health perceptions among medical students.

Manuscript Change:

Discussion; pp. 18; lines 339-342.

- **Comment R2.3 Acknowledge unmeasured confounding**

Response: Additional text has been added to the Discussion acknowledging that cultural norms, latitude/season, income, and curricular differences may influence behaviors and should be measured in future studies.

Manuscript Change:

Discussion → Limitations; pp. 21; lines 411-414.

- **Comment R2.4 Conclusions**

Response: We updated the Conclusion to more cautiously frame results as hypothesis-generating, recommending future multicenter studies and curriculum-aligned interventions.

Manuscript Change:

Conclusions; pp. 19-20; lines 367-394.

Finally, we thank the Reviewers and Editorial Team again for their valuable comments. We believe the manuscript has been substantially improved.

VERSION 2 - REVIEW

**Reviewer**	**2**
**Name**	**Hashizume, Hideo**
**Affiliation**	**Shimada Municipal Hospital**
**Date**	**08-Dec-2025**
**COI**	

This has been well revised according to my comments.
